# De Novo Donor-Specific Antibodies after Heart Transplantation: A Comprehensive Guide for Clinicians

**DOI:** 10.3390/jcm12237474

**Published:** 2023-12-02

**Authors:** Irene Marco, Juan Carlos López-Azor García, Javier González Martín, Andrea Severo Sánchez, María Dolores García-Cosío Carmena, Esther Mancebo Sierra, Javier de Juan Bagudá, Javier Castrodeza Calvo, Francisco José Hernández Pérez, Juan Francisco Delgado

**Affiliations:** 1 Cardiology Department, Hospital Universitario La Paz, 28046 Madrid, Spain; irene.marco@salud.madrid.org; 2 Cardiology Department, Hospital Universitario Puerta de Hierro, 28222 Madrid, Spain; juancl09@ucm.es (J.C.L.-A.G.); fhernandezp@salud.madrid.org (F.J.H.P.); 3 Centro Nacional de Investigaciones Biomédicas en Red de Enfermedades CardioVasculares (CIBERCV), 28029 Madrid, Spain; javier.gonzalezm@salud.madrid.org (J.G.M.); mariadolores.garcia-cosio@salud.madrid.org (M.D.G.-C.C.); javier.juan@salud.madrid.org (J.d.J.B.); jcastrodeza5@gmail.com (J.C.C.); 4 School of Medicine, Universidad Europea de Madrid, 28670 Madrid, Spain; 5 Cardiology Department, Hospital Universitario 12 de Octubre, Instituto de Investigación Sanitaria Hospital 12 de Octubre (imas12), 28041 Madrid, Spain; andrea.severo@salud.madrid.org; 6 Immunology Department, Hospital Universitario 12 de Octubre, Instituto de Investigación Sanitaria Hospital 12 de Octubre (imas12), 28041 Madrid, Spain; esther.mancebo@salud.madrid.org; 7 Cardiology Department, Hospital Universitario Gregorio Marañón, 28007 Madrid, Spain; 8 School of Medicine, Universidad Complutense de Madrid, 28040 Madrid, Spain

**Keywords:** heart transplantation, donor-specific antibody, late graft dysfunction, antibody-mediated rejection, immunosuppression

## Abstract

Antibodies directed against donor-specific human leukocyte antigens (HLAs) can be detected de novo after heart transplantation and play a key role in long-term survival. De novo donor-specific antibodies (dnDSAs) have been associated with cardiac allograft vasculopathy, antibody-mediated rejection, and mortality. Advances in detection methods and international guideline recommendations have encouraged the adoption of screening protocols among heart transplant units. However, there is still a lack of consensus about the correct course of action after dnDSA detection. Treatment is usually started when antibody-mediated rejection is present; however, some dnDSAs appear years before graft failure is detected, and at this point, damage may be irreversible. In particular, class II, anti-HLA-DQ, complement binding, and persistent dnDSAs have been associated with worse outcomes. Growing evidence points towards a more aggressive management of dnDSA. For that purpose, better diagnostic tools are needed in order to identify subclinical graft injury. Cardiac magnetic resonance, strain techniques, or coronary physiology parameters could provide valuable information to identify patients at risk. Treatment of dnDSA usually involves plasmapheresis, intravenous immunoglobulin, immunoadsorption, and ritxumab, but the benefit of these therapies is still controversial. Future efforts should focus on establishing effective treatment protocols in order to improve long-term survival of heart transplant recipients.

## 1. Introduction

HLA (human leukocyte antigen) molecules are a group of highly polymorphic antigens encoded by the major histocompatibility complex. Their role is to present short peptides from the intracellular and extracellular compartments to T lymphocytes. All nucleated cells express HLA class I on their surface, whereas HLA class II is only expressed on antigen-presenting cells [1]. HLA molecules play a key role on the human immune system, enabling the recognition of “non-self” antigens and activation of defense mechanisms [2]. However, their polymorphism represents a threat to graft survival in the field of transplantation. HLA sensitization refers to the development of antibodies directed against foreign HLAs. In the case of transplant recipients, these antibodies can be directed against donor antigens, known as donor-specific antibodies (DSAs). DSAs are not exclusively anti-HLA antibodies, and evidence about antibodies directed against other donor antigens has also emerged during recent years.

Anti-HLA DSAs have consistently been associated with worse outcomes in solid organ transplantation [3,4]. DSA can develop before transplantation (preformed DSA) or appear de novo afterwards (de novo DSA, dnDSA). Transplantation in the presence of preformed DSA is related to worse post-HT outcomes [5], and thus, preformed antibodies act as a limitation for transplant by reducing the number of compatible donors. However, it is dnDSAs that are considered a major threat to transplant recipients [6,7]. In patients with heart transplant (HT), they have been associated with antibody-mediated rejection (AMR) [6,8], cardiac allograft vasculopathy (CAV) [9,10], graft dysfunction [11], and mortality [12,13,14]. They represent one of the main concerns of the HT scientific community because of their tight relationship with long-term outcomes, which have remained barely unchanged over recent years [15]. Development of solid-phase assays has meant a giant leap for the field, as they have permitted precise DSA detection and identification. Their expanding use has enabled us not only to broaden our knowledge about antibody significance but also to implement routine monitoring protocols in most HT teams [16]. It has become a powerful non-invasive tool to aid in the surveillance of HT recipients. However, the increasing body of evidence concerning dnDSA has also brought new questions to the table. The chronology of dnDSA injury on the graft, the best approach to dnDSA management in the absence of detectable graft injury, or the real efficacy of current treatments are some of the issues under study. In this article, we will review the existing evidence about dnDSA with the aim of providing a helpful framework for clinicians to help decision making and future investigations.

## 2. Mechanisms of dnDSA Development and Pathogenicity

De novo DSAs (dnDSAs) are defined as new DSA appearing more than 3 months after transplant and are considered an alloimmune primary response [17], in contrast to preformed DSAs, which develop prior to transplant. DSAs newly detected during the first 3 months post-HT are also considered preformed [17], as they reflect alloimmune memory, where re-exposure triggers a recall response in a pre-sensitized patient [18]. Sensitization occurs after exposure to “non-self” HLA during pregnancy, blood transfusions, or transplantation, but also after an HLA-unrelated immune stimulus, probably secondary to cross-reactivity between pathogens and HLA [19]. However, not all sensitizing events lead to the development of antibodies. It is theorized that alloimmunization requires a “double hit” from a non-self stimulus (foreign HLA) and a danger stimulus such as surgery, tissue injury, or other inflammation states [20,21,22]. Antibodies are directed against epitopes, which are hypervariable regions on HLA molecules. One antibody can react against several antigens, as the same epitope can appear on several HLA molecules [2].

DSAs exert their pathogenic effects at the level of the graft endothelium, which acts as the interface between the receptor’s circulating blood and the donor’s tissue. Tissue damage during transplantation induces inflammation and secondarily promotes HLA class II expression on endothelial cells [23]. After DSA binds to endothelial HLA, the activation of the classical complement cascade ultimately leads to the membrane attack complex formation and cell lysis by lymphocytes. Accordingly, AMR biopsies typically test positive for C4d, a component of the classical complement pathway [20]. However, DSA can also damage the graft without complement activation via antibody-dependent cell-mediated cytotoxicity by innate immune cells such as natural killer cells. This mechanism could explain C4d-negative antibody-mediated rejection and other DSA effects such as microvascular injury [24,25]. Finally, DSA binding to endothelial cells activates signaling pathways responsible for intimal proliferation and fibrosis seen in chronic AMR [20,23]. T-helper cells play a central role in the immune response caused by DSA binding, as they are responsible for the activation and regulation of other immune and non-immune cells. Instead of describing T-helper cells based on their cytokine profile, a novel approach is to define them based on the cells they help. T-helper cells can provide help to mononuclear phagocytes (Type 1), to B cells and polymorphonucleated granulocytes (Type 2), or to non-immune tissue cells, such as endothelial graft cells (Type 3) [26]. This triple classification helps to better understand the immunological processes observed in transplantation, regenerative medicine, and tissue engineering [27].

As McCaughan et al. describe, preformed DSA and dnDSA may damage heart allografts in different ways. Preformed DSAs act early after transplantation at a time when patients are closely monitored. Graft injury depends on complement activation and antibody-dependent cell-mediated cytotoxicity, and early treatment can prevent chronic damage before other immune cells are involved. On the other hand, dnDSA development implies a deeper activation of the immune system, with an inflammatory event prompting the expression of mainly HLA class II on the graft. In this setting, the production of antibodies requires de novo B cell activation and formation of plasma cells, and results in the implication of innate immune cells at a time when surveillance is relaxed. In this setting, dnDSAs can cause extensive damage before they are clinically detected, with treatments being less effective at this point [20,28].

## 3. DnDSA Detection Methods

Solid-phase single-antigen bead (SAB) assays on the Luminex platform are nowadays the standard of care for dnDSA detection [16]. Before performing a SAB assay, many laboratories perform a screening test with pooled antigen panels that detect the presence of class I or class II anti-HLA antibodies without providing HLA specificity [29]. In the SAB assay, multiple fluorochrome-infused beads coated with individual HLA molecules are exposed to the recipient’s serum. Anti-HLA antibodies bound to their corresponding HLA molecules subsequently bind to anti-Ig G antibodies labeled with a fluorescent dye. Beads are analyzed by a dual laser that detects both the specific bead and the presence of the bound antibody [20]. Results are reported as mean fluorescent intensity (MFI) for each anti-HLA antibody. MFI values are semiquantitative and should be interpreted as such: an approximated value of the amount of antibody [30]. 

SAB assays have meant a revolution in the field of solid organ transplantation as they yield the highest sensitivity and resolution. HLA typing methods have also improved over the years, and both advances have enabled the precise identification of antibody specificity and the detection of DSA [31]. In order to further stratify the risk of DSA, laboratories also use modifications of the SAB technique that evaluate complement binding (C1q, C3d, C4d) or Ig G subclasses. Complement-fixing DSAs have been related to an increased risk of allograft rejection and worse survival compared to non-complement-fixing DSAs [32,33,34]. Evidence on the differential effects of Ig G subclasses, with complement-binding IgG1 and IgG3 being more deleterious than IgG2 and IgG4, is more controversial [33,35]. Over recent years, there has been a growing interest for non-HLA antibody testing, as they could be responsible for AMR with no detectable DSA. Nowadays, many laboratories also test for the presence of non-anti-HLA antibodies such as anti-MICA/B (MHC class I polypeptide-related sequence A/B), anti-endothelial, anti-vimentin, or angiotensin-1 receptor antibodies [16].

A correct interpretation of Luminex results provides highly valuable information in the assessment of patients with dnDSA in two main settings: (a) risk stratification of newly detected dnDSA, in order to guide management, and (b) evaluation of treatment efficacy. However, SAB assays convey a number of limitations that the clinician needs to acknowledge (Table 1). Misinterpretation of these tests is not uncommon, with MFI erroneously considered a quantitative value. Also, techniques and MFI thresholds are not homogenously standardized between laboratories, which means lower reproducibility of the results. Finally, the high sensitivity of SAB assays can sometimes be detrimental, as detected antibodies can be clinically irrelevant, prompting the start of unnecessary and potentially harmful treatments. In that sense, effective communication between immunologists and clinicians is imperative in order to obtain the most clinically relevant information.

## 4. DnDSA Incidence and Monitoring in Heart Transplantation

The International Society for Heart and Lung Transplantation (ISHLT) consensus document from 2018 and the 2022 ISHLT Guidelines for the Care of Heart Transplant Recipients recommend post-HT monitoring for dnDSA at 1, 3, 6, and 12 months post-HT and then annually in low-risk patients and more frequently in sensitized patients [16,37].

As the consensus states, the choice of this protocol is based on the transient character of some dnDSAs and the belief that early antibodies may be more easily treated. However, there are no data about the efficacy of this screening protocol and the optimal monitoring periodicity remains unknown. Most studies about dnDSAs in HT focus on their prognostic implication, with less information concerning the chronology of dnDSA development and their persistence over the years. Table 2 summarizes the available evidence concerning dnDSA epidemiology gathered from studies based on SAB assays and including adult patients. Studies are generally retrospective and differ in MFI thresholds, frequency of determinations, follow-up times, and definition of persistent dnDSA.

Prevalence of dnDSAs after HT varies from 10 to 30%, with dnDSAs directed against HLA class II being more frequent than dnDSAs directed against HLA class I or both classes [6,8,10,11,12,13,14,28,38,39,40]. Few studies have reported dnDSA incidence over time, with median time to dnDSA development ranging from less than a year [13,40] to more than 7 years in the study with the longest follow-up [10]. Even if the highest incidence of dnDSA development seems to occur during the first year [41], dnDSAs have been reported to develop as late as 19.5 years after HT [42]. This evidence supports maintaining anti-HLA screening indefinitely; however, almost a third of HT groups stop screening after the first year post-HT [16]. DnDSAs directed against HLA-DQ appear to be the most frequent dnDSA and tend to develop later and to be more frequently persistent than other dnDSAs [8,11,12,28,38,40].

The 2018 ISHLT consensus document recommends individualizing DSA monitoring depending on the patient’s risk, but only considering sensitized patients as high-risk patients. Actually, there is a paucity of data concerning risk factors in HT patients, and most evidence comes from studies in renal transplantation. Pre-sensitized patients with preformed DSA or non-donor-specific preformed anti-HLA antibodies appear to have a higher risk for dnDSA development than non-sensitized patients [39,43,44]. However, dnDSA development seems to occur earlier than in non-sensitized patients [28], and may respond to an activation of memory B cells rather than naïve B cells and therefore differ from late dnDSA. In that sense, it seems reasonable to keep a closer surveillance on sensitized patients, but it could be limited to the first year post-HT. Table 3 summarizes other described risk factors for dnDSA development. There is little information about the impact of different immunosuppression regimens on dnDSA production. Early corticosteroid withdrawal seems safe, as it did not increase the risk of dnDSA development in a randomized trial of kidney transplant recipients [45] nor in a retrospective study of 229 HT patients [46]. An early switch from calcineurin inhibitor to mechanistic target of rapamycin (mTOR) inhibitor monotherapy may be associated with a higher risk of dnDSA appearance, but late conversion or the combination of mTOR inhibitor with reduced-exposure calcineurin inhibitor appears to be safe [47]. Pro-inflammatory events such as infections, vaccines, or surgeries may trigger a more unspecific immune response, as they increase the production of anti-HLA antibodies [48,49], but not of DSA. Finally, left ventricular assist devices have been associated with a higher risk of developing anti-HLA antibodies pre-HT [50], but there is no evidence about them conferring a higher risk of developing dnDSA.

## 5. Clinical Implications of dnDSA Development in HT Recipients

### 5.1. DnDSA and Survival

DSAs have consistently been associated with worse survival after HT [9,12,13,14,39], but this is particularly true for dnDSA. In the study by Clerkin et al., patients with dnDSA had a 151% increase in graft loss (defined as mortality or retransplantation) compared with non-DSA patients [6]. It is important to note that poor survival is related to dnDSA but not de novo non-DSA anti-HLA antibodies [12]. Evidence also shows that the greatest impact on mortality comes from persistent dnDSA, whereas transient dnDSA may not have an impact on mortality [12,13]. However, definitions of dnDSA persistence vary between studies. Late antibodies also appear to confer higher mortality risk than antibodies detected during the first year post-HT [7]. The impact of complement-fixing dnDSA on mortality is less evident. Smith et al. found no increased risk in mortality of complement-fixing dnDSA using C3d SAB assay and neither did Farrero Torres et al. using C1q SAB assay [12,39]. However, a retrospective study of 282 patients did find a worse survival in patients with C3d-fixing dnDSA compared to patients with non-C3d-fixing dnDSA [10]. Finally, dnDSA specificity may also have an impact on mortality, with worse survival associated with dnDSA against HLA class II, and not against HLA class I [6]. Also, Cole et al. described a 6-fold higher risk of death in patients with anti-HLA-DQ dnDSA compared to patients with other dnDSA and non-dnDSA patients [11].

### 5.2. DnDSA and Cardiac Allograft Vasculopathy

Evidence concerning dnDSA association with CAV is also robust. The majority of studies have reported a higher incidence of CAV in patients with DSA [9,54,55]. Smith et al. found no correlation between DSA and CAV, but when the causes of death were analyzed, more patients with DSA died of CAV or rejection than patients without DSA [12]. These differences are probably related to the complex pathophysiology of CAV development, which combines both immune and non-immune mechanisms. The contribution of non-immune mechanisms such as donor and recipient age and cardiovascular risk factors could obscure CAV’s association with dnDSA. Furthermore, dnDSA impact on cardiac vasculature seems to be progressive and may take months or even years [56]. In the study by Kaczmarek et al., Kaplan–Meier curves for freedom from CAV separated after roughly six years post-HT [9]. Late dnDSAs (detected more than one year after HT) appear to lead to a higher risk of CAV than early dnDSA [57], and so do complement-fixing DSAs, emphasizing again the more aggressive nature of complement-fixing antibodies [10,58]. The study by Wang et al. did not differentiate between de novo and preformed DSA, but found persistent, 1:8 dilution, C1q-positive and class II DSA to be associated with more severe CAV [59].

### 5.3. DnDSA, Antibody-Mediated Rejection, and Graft Failure

Although DSAs are believed to be the cause of AMR, their presence is not required for its diagnosis in HT, according to the ISHLT grading system. Indeed, AMR diagnosis is based purely on pathologic criteria, without accounting for DSA presence or graft failure [60]. This controversial decision was meant to standardize AMR diagnosis in research works until antibody determinations were more consistent among HT groups [61]. Even if DSAs offer a high sensitivity and specificity for AMR diagnosis, they are not detectable in all cases, as not all relevant antibodies have been identified yet [16]. On the other hand, accommodation to DSA has been described, where in some cases, complement deposition does not lead to graft dysfunction since regulatory proteins achieve the termination of the complement cascade [62]. Complement-fixing DSA may offer a higher predictive value for AMR [32,39,63]. Also, some studies have tried to find an MFI threshold that may improve AMR prediction. However, results differ substantially, probably because of MFI being a semiquantitative value [64,65]. 

However, neither are pathological findings fully reliable to confirm AMR, as there is a high variability in histopathologic interpretation [66]. In that sense, dnDSAs are a highly valuable tool to interpret dubious pathological findings. Moreover, they certainly provide prognostic information. Clerkin et al. reported that DSA detection during an episode of AMR was associated with a 5-fold increased risk of graft dysfunction. Also, detection of DSA against HLA class II in patients without AMR was associated with a 3-fold increase in the odds of future AMR [6]. These results suggest that DSA may be a marker of subclinical graft damage even in the absence of AMR by current pathologic criteria. 

**Figure 1:**
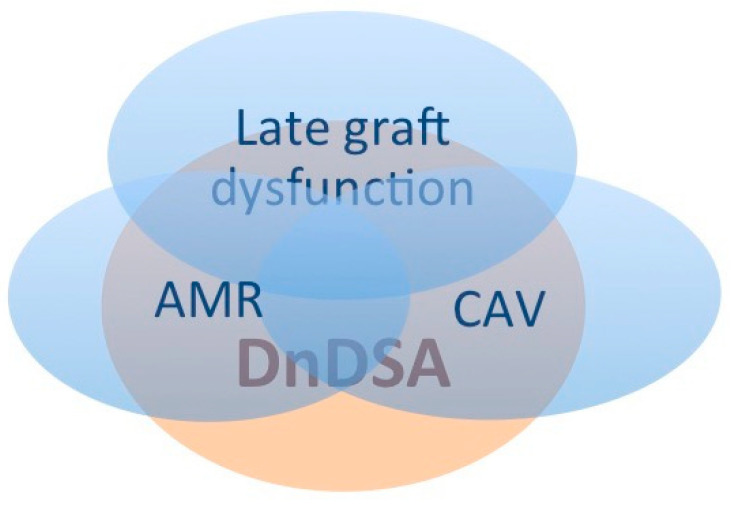
Association between late graft dysfunction, AMR, CAV, and dnDSA. AMR: antibody-mediated rejection; CAV: cardiac allograft vasculopathy; dnDSA: de novo donor-specific antibody.

The definition of late cardiac allograft dysfunction varies widely among clinical studies, illustrating the fact that the underlying mechanisms are still poorly understood. However, it is one of the main causes of long-term mortality after HT and one of the central concerns of HT teams [67,68,69]. Late graft dysfunction refers to a situation of chronic cardiac failure, where the main feature is a restrictive physiology, and usually left ventricular function is preserved [67,70]. Growing evidence points towards a strong association between late graft dysfunction, late chronic AMR, and CAV, with dnDSAs being the connecting link between the three (Figure 1) [71,72,73]. Whereas AMR was previously considered an early event occurring mainly in patients with preformed DSA, recent studies show that late AMR in the setting of dnDSA is a distinct entity and portends a dreadful prognosis. Hodges et al. studied fifteen patients with late AMR and dnDSA. The median time from HT to AMR was 4.5 years and persistent cardiac dysfunction developed in 46% of patients, with a median survival of 0.8 years [71]. In another study including twenty patients with treated late AMR occurring at least one year after HT, eight patients died within 3 months and a third of the remaining patients developed persistent left ventricular dysfunction. Of note, fulminant CAV was reported in 17% of survivors [72].

## 6. Evaluation of Patients with dnDSA

Given the above-mentioned association of dnDSA with mortality, AMR, and CAV, routine monitoring of anti-HLA antibodies represents an invaluable tool for the long-term surveillance of HT recipients. Along with other non-invasive biomarkers, HLA antibodies enhance risk stratification of HT patients so that the frequency of endomyocardial biopsies can be reduced [74,75].

After dnDSA detection, the clinician should undertake a careful evaluation of the graft function. Echocardiography can show signs of diastolic dysfunction, which may be an early sign of rejection but is generally unspecific [76,77]. Endomyocardial biopsy should be performed, especially in the presence of dnDSA with high-risk features, as it is the gold standard for AMR diagnosis [78]. Description of AMR should be made following the ISHLT grading system, which is based on histopathologic and immunohistochemical criteria [60]. However, the clinician should bear in mind that AMR pathological diagnosis may be challenging even following strict criteria; discordance between pathologists was shown to be non-negligible in the setting of acute cellular rejection, where diagnostic criteria are better defined [66]. Finally, performing a coronary angiogram is advisable given dnDSA association with CAV.

In some cases, evaluation will find no evidence of AMR, CAV, or graft dysfunction. However, these complications may arise years after dnDSA detection with a higher incidence than in non-dnDSA patients [6]. Therefore, what we are lacking is effective instruments to detect subclinical dnDSA damage. Some promising diagnostic tools have emerged in recent years, most of them with the additional advantage of being non-invasive. Donor-derived cell-free DNA (dd-cfDNA) could be of great value for identifying dnDSA patients at higher risk, as levels of dd-cfDNA have been shown to increase in the setting of AMR [79]. Gene expression profiling could also provide valuable information by defining specific rejection phenotypes that could help tailor treatments and measure therapeutic responses [4,80]. In echocardiography, global left ventricular strain and right ventricular free wall longitudinal strain have shown promising results in ruling out acute cellular rejection, so they could have a role in detecting subclinical AMR [81]. Cardiac magnetic resonance may offer valuable information about myocardial edema and fibrosis in dnDSA patients. Indeed, increased T2 recovery times combined with elevated ECV have been associated with acute rejection [82]. Regarding pathologic assessment, the use of electron microscopy has improved the detection of endothelial damage to the point of being included in the diagnostic criteria of kidney AMR. Electron microscopy enables the evaluation of early endothelial lesions such as endothelial cell enlargement or capillary basement membrane multilayering [83]. However, further studies are needed in order to assess its utility in cardiac grafts. As for CAV diagnosis, intravascular ultrasound can detect intimal thickening before angiographic lesions are visible [84] and invasive assessment of coronary physiology with fractional flow reserve and microcirculatory resistance may also help individualize risk in dnDSA patients [85].

## 7. Current Management of dnDSA

In current practice, several therapeutic options are available to treat dnDSA and AMR, although none have received FDA approval [4]. Evidence on efficacy comes mainly from studies of desensitization in the pre-transplant setting or from the field of renal transplantation, with limited information concerning their impact in HT. The objective of treatment is not only to remove circulating antibodies and to block their effects, but also to suppress their production. To achieve both purposes, centers generally use a combination of agents that target different pathophysiologic pathways. Table 4 summarizes the mechanisms of action of the available therapies. 

Extracorporeal treatments include plasmapheresis, immunoadsortion, and photopheresis. Plasmapheresis results in antibody removal by extracting plasma volume from the patient and replacing it with exogenous albumin or plasma. Immunoadsortion does not need to replace fluids, as it specifically removes immunoglobulins; however, it is more costly, less widely available, and less efficient in cytokine removal [78,94]. Double filtration plasmapheresis is a novel technique that may present the advantage of not only removing antibodies but also complement factors [95]. Plasmapheresis and immunoadsortion are inefficient by themselves, as they only remove immunoglulins and cytokins from the vascular space, which eventually equilibrates with the interstitium, needing multiple sessions and the use of additional immunosuppressive agents [2]. As for photopheresis, it is a leukapheresis technique where lymphocytes are extracted, radiated, and subsequently reinfused into the patient. Their apoptosis induces immunomodulatory effects on T cells, so that multiple sessions could have a role in chronic AMR [96,97]. 

IVIG, a polyclonal IgG preparation from pooled human plasma, was first used for the treatment of immunodeficiency disorders. However, its use expanded quickly to the treatment of autoimmune and inflammatory diseases because of its immunomodulatory and anti-inflammatory effects at high doses. Although incompletely understood, IVIG’s role in AMR is based on complement inhibition, expansion of T-regulatory cell populations, saturation of fragment cystallizable (Fc) receptors, and neutralization of autoantibodies and cytokines [98,99]. 

Plasmapheresis or immunoadsortion sessions combined with IVIG cycles are considered the standard of care for acute AMR in renal transplantation [100]. Small studies support their benefits in short-term graft survival [101,102], although their long-term effects remain uncertain. The majority of protocols for AMR treatment include corticosteroid pulses because of their previous widespread use in acute cellular rejection. Although their strong immunosuppressive effects are well-known, there are no reliable data about their impact on dnDSA [78]. Antithymocyte globulin has also been adopted from acute cellular rejection therapeutic schemes and is used as a potent cytolytic therapy when hemodynamic compromise is present [103]. 

Numerous transplant centers add rituximab to their treatment strategies with the aim of suppressing memory B cells and thus improving long-term outcomes. Rituximab, an anti-CD20 monoclonal antibody, showed normalization of ventricular function and resolution of AMR in a small series of eight HT patients when used in monotherapy [104]. However, no clear benefit was observed in a randomized trial of kidney transplant patients, although the study was underpowered and patients were also receiving adjunctive therapies [86].

Some centers prefer to target plasma cells by using proteasome inhibitors such as bortezomib or carfilzomib. Bortezomib was first used in multiple myeloma and has shown controversial results in renal transplantation, with a recent randomized trial showing no benefit in patients with late AMR and DSA [105]. Other monoclonal antibodies are being studied to treat chronic refractory AMR caused by persistent dnDSA. Complement inhibition by anti-C5 eculizumab may be a promising therapeutic resource. A randomized study suggested a benefit in preventing acute AMR in sensitized kidney transplant recipients [87], and recently, Coutance et al. published the intermediate-term outcomes of eculizumab use in highly sensitized recipients, showing a non-significant lower incidence of pAMR2-3 and left ventricular dysfunction [88]. The use of C1 esterase inhibitors is also under study with encouraging results [89]. Another interesting approach may be to block IL-6, a pro-inflammatory cytokine involved in atherosclerotic progression and graft rejection, with the use of tocilizumab or clazakizumab [90,91]. Alemtuzumab, an anti-CD52 monoclonal antibody that suppresses mature lymphocytes, has also been used in case reports of refractory rejection [92]. Finally, the use of other agents such as belimumab (anti-B-lymphocyte stimulator monoclonal antibody) or daratumumab (anti-CD38 monoclonal antibody) has been described in case reports with positive results [93,106]. However, the potential toxicity of these agents must be borne in mind before more solid evidence about their benefits is available. 

Given the scarcity of data, it is difficult to establish clear recommendations for when to treat dnDSA. There is a general agreement that dnDSA should be treated in the presence of graft dysfunction and restrictive physiology and/or in the presence of AMR [16,78], but other scenarios are controversial. It seems reasonable that the first step after detecting dnDSA should be to optimize immunosuppression by targeting higher levels of CNI, increasing antimetabolite dose or adopting a regimen with mTOR, particularly if CAV is also detected. In the 2018 ISHLT consensus document, most participants declared they would not treat DSA in the absence of graft dysfunction but would increase surveillance, although a minority would consider treating complement-fixing or high-level DSA. 

**Figure 2:**
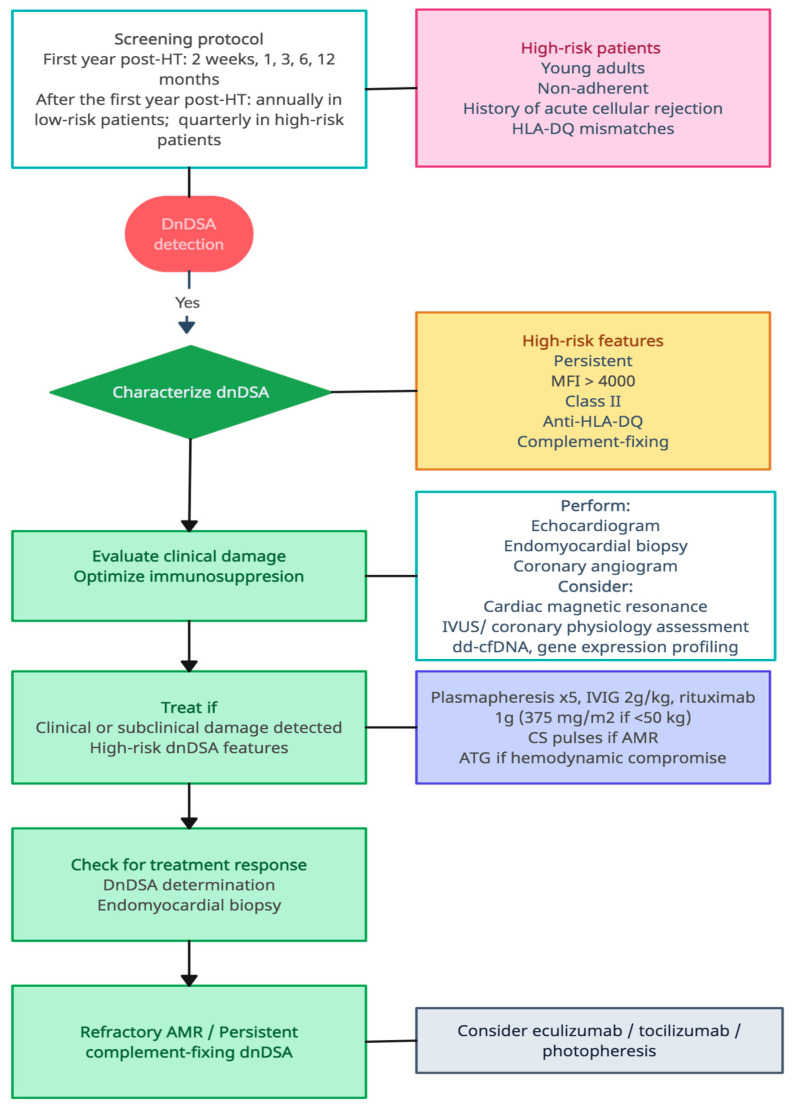
Proposed algorithm for dnDSA management. HT: heart transplant; MFI: mean fluorescence intensity; dnDSA: de novo DSA; AMR: antibody-mediated rejection; dd-cfDNA: donor-derived cell-free DNA; ATG: antithymocyte globulin; IVIG: intravenous immunoglobulin; CS: corticosteroids.

Figure 2 shows our proposed algorithm for management of dnDSA. We advocate for a proactive approach with special emphasis in dnDSA regular screening, particularly in patients at high risk of developing dnDSA. DnDSA detection should be followed not only by a search for evidence of graft injury but also by a careful characterization of antibodies, as it provides essential information about their pathogenic potential. In the presence of dnDSA with high-risk features, clinicians should not be satisfied with the absence of AMR, CAV, or echocardiographic graft dysfunction, and they should look for evidence of subclinical damage with the aid of other diagnostic tools such as cardiac magnetic resonance, biomarkers, or other invasive coronary techniques. Treatment should always be started when damage is observed, but it might also be considered in the presence of persistent, class II dnDSA with high MFI, especially anti-DQ dnDSA, even in the absence of graft injury [18,75]. Our treatment scheme includes five sessions of plasmapheresis or immunoadsortion followed by IVIG infusion and one to four doses of rituximab; additionally, corticosteroids should be considered when AMR is present and ATG if there is hemodynamic compromise. Another key issue is how to monitor treatment response. In our opinion, both a negative endomyocardial biopsy and the negativization of dnDSA should be pursued, as the presence of persistent complement-fixing dnDSA after AMR treatment has been associated with worse long-term outcomes in several studies in renal transplantation [107,108]. Finally, clinicians may consider the use of other monoclonal antibodies or photopheresis in the presence of refractory AMR or persistent high-risk dnDSA; however, response to treatment is less probable when graft injury becomes chronic. 

## 8. Future Perspectives

The importance of dnDSA should never be underestimated, as it may determine a patient’s long-term survival. Mechanistic studies are needed to better understand the continuum of dnDSA chronic effects on cardiac allografts. This information will enable the development of diagnostic tools capable of detecting subclinical graft injury where current methods fail [109]. Surely myocardial mapping with cardiac magnetic resonance could be a promising resource to detect graft injury when dnDSAs are detected but pathological findings are inconclusive. 

Further investigations on non-HLA antibodies are also of great interest to better characterize AMR cases where anti-HLA dnDSAs are negative. There is a need to develop Luminex assays to detect non-HLA antibodies simultaneously to anti-HLA antibodies, as they may provide complementary information [110]. 

Clinicians will probably become more aggressive in dnDSA management once reliable evidence about treatment efficacy is obtained from randomized trials. Biological agents may enable targeted treatments tailored to dnDSA characteristics. Valuable information may be extrapolated from clinical trials conducted in renal transplantation, where investigations in dnDSA and AMR are usually one step ahead. For example, a large placebo-controlled international clinical trial is testing clazakizumab in chronic AMR [111]. Similar efforts should be undertaken in HT recipients.

Despite the importance of advances in diagnostic and treatment strategies, the optimal approach to improve long-term outcomes most certainly relies on preventing dnDSA appearance. In that sense, tools that could predict dnDSA development would be extremely useful. Elevations in dd-cfDNA and in gene expression profiling have been associated with subsequent dnDSA detection [112,113,114]. Although a causal relationship has not been established, dd-cfDNA or gene expression profile monitoring could help individualize immunosuppression regimens. In the near future, post-transplant surveillance will probably rely on a global assessment of the patient’s immune state by combining genomic and proteomic information obtained from new molecular technologies [74]. More studies are needed to further analyze the association between dnDSA and microRNA, gene and protein expression profiling, and dd-cfDNA.

## Figures and Tables

**Table 1:** Major limitations of SAB essays [16,29,30,35,36].

Limitation	Effect	Potential Solutions
Denatured antigens: distortion of HLA molecules bound to the solid matrix exposes antigens not present in vivo	False positives	Use of cell-based assays to test clinical relevance
Saturation: the finite amount of HLA molecule on a bead is saturated by antibody binding, with the overload being undetected	Underestimation	Serum dilutions
Complement interference: activation of the complement cascade prevents binding of the detection antibody	Underestimation	Serum dilutions, use of EDTA or DTT pretreated or plasma samples
Cross-reactive epitopes: the same antibody binds to HLA molecules on different beads	Underestimation	Identifying specific reactive patterns belonging to epitopes shared by several HLA molecules
Inter- and intra-laboratory variability	Under/overestimation, low reproducibility	Standardized protocols, quality control procedures, high expertise
Treatment with polyclonal ATG: rabbit antibodies are detected as human antibodies or compete with them for binding to HLA molecules	False positives/underestimation	
Treatment with IVIG: high doses can increase background fluorescence	Problematic test interpretation	

SAB: single-antigen bead; HLA: human leukocyte antigen; ATG: antithymocyte globulin; IVIG: intravenous immunoglobulin.

**Table 2:** Prevalence, incidence, and characteristics of DnDSA reported in literature.

Study (First Author, Year)	N	DnDSA Determinations Post-HT	Laboratory Tests	DnDSA Incidence and Characteristics
Smith, 2011 [12]	243	Annually when possible Maximum FU 13 years	SAB, C4d SAB Threshold MFI > 1000	DnDSA: 25.4% (57/224) Against class I: 8; class II: 37; both classes: 12 Anti-HLA-DQ most frequent DSA Persistent dnDSA: 48/57 (C4d+: 26) All dnDSA within 8 years post-HT
Reinsmoen, 2014 [38]	200	5 during 1st year when possible	SAB, Threshold not stated	DnDSA: 9.5% (19/200) Against class I: 2; class II: 12; both classes: 5 Anti HLA-DQ more frequent
Clerkin, 2017 [6]	221	Frequently during 1st year, yearly thereafter Median FU 3.5 years	SAB, any MFI	DnDSA: 24% (53/221)
Cole, 2017 [11]	122	2 weeks, 1, 3, 6, 12 months, yearly thereafter Mean FU 3.3 years	SAB, Threshold MFI > 1000	DnDSA: 28% (31/122) Anti-HLA-DQ: 19 (later post HT, more frequently persistent and with higher MFI) Mean time to dnDSA detection: 539 days
Farrero Torres, 2017 [39]	125	Quarterly during 1st year, at clinical request thereafter	SAB, C1q SAB Threshold MFI > 999	Preformed DSA: 20.1% (29/144), 4 without DSA post-HT were excluded DnDSA: 39.7% (48/121) Median time to dnDSA detection: 232 days for C1q-, 396 days for C1q+
Zhang, 2018 [8]	176 (A/P)	Frequently during 1st year, quarterly thereafter Median FU 16–18 months	SAB, C3d SAB Threshold MFI > 1000 for HLAA, B, DR, DQ, > 2000 for HLA C, DP	Preformed DSA: 12.5% (22/176) DnDSA: 29.7% (43/154) Against class I: 6; class II: 24; both classes 13 Anti-HLA-DQ most frequent dnDSA C3d+ DSA: 21.5% (14/65) of all DSA 3-year cumulative incidence 28%
McCaughan, 2018 [40]	240	Several determinations during 1st year, yearly thereafter Median FU 1496 days	SAB, Threshold MFI >1200	DnDSA: 27% (24/240) Anti HLA-DQ: 36 Persistent dnDSA: 38 (27 anti-HLA-DQ) Median time to dnDSA detection: 308 days
Moayedi, 2018 [13]	179 (A/P)	1, 3, 6, 12 months, yearly thereafter; quarterly if DSA Median FU 4.1 years	SAB, Threshold MFI > 1200	DnDSA: 23% (42/179) Persistent dnDSA: 27 (21 anti HLA-DQ) Median time to dnDSA detection: 329 days
Zhang, 2020 [28]	548	1, 3, 6, 12 months, yearly thereafter Median FU 805 days	SAB Threshold MFI > 2500	Preformed DSA: 6.2% (34/548) DnDSA: 12% (63/514) Anti-HLA-DQ most frequent dnDSA, detected later than HLA-A/B Time to dnDSA generally <2000 days
Baudry, 2022 [10]	282	4 during 1st year; 1 at the time of the study; if +, previous annual samples were analyzed Median FU 14.3–16.4 years	Luminex screening assay, if positive SAB and C3d SAB Threshold MFI > 500	Sensitized patients excluded DnDSA: 18.1% (51/282) C3d+ dnDSA: 29 Median time to dnDSA detection: 7.7 years for C3d−, 10.1 years for C3d+
Akhtar, 2023 [14]	232	1, 3, 6, 12 months, yearly thereafter Median FU 4.7 years	SAB Threshold MFI > 1000	DnDSA 23.7% (55/232) Against class II: 54 Anti HLA-DQ most frequent dnDSA All dnDSA within 9.5 years post-HT

N: number of patients; A/P: adult and pediatric patients; FU: follow-up; DSA: donor-specific antibodies; dnDSA: de novo DSA; SAB: single-antigen bead; HLA: human leukocyte antigen; HT: heart transplant; MFI: mean fluorescence intensity.

**Table 3:** Described risk factors for dnDSA development in the literature.

Risk Factors for dnDSA Development
Pre-sensitization [34,39,43]
Younger age in adult recipients [9,39,41,51]
Older age in pediatric recipients [42]
HLA mismatches (particularly HLA-DQ mismatches) [20,28,52]
Episodes of acute cellular rejection during the first year post-HT [12,51]
Non-adherence, suboptimal immunosuppression [51,53]

DnDSA: de novo DSA; HLA: human leukocyte antigen; HT: heart transplant.

**Table 4:** Mechanism of action of therapies used for dnDSA management [78,86,87,88,89,90,91,92,93].

Mechanism of Action	Therapies
Removal of circulating antibodies	Plasmapheresis, immunoadsortion
Inhibition of auto-antibody effects	IVIG
Depletion of B cells	Corticosteroids, rituximab, belimumab, alemtuzumab
Depletion of plasma cells	Bortezomib, carfilzomib, daratumumab
Suppression of T-cell response	Corticosteroids, ATG, photopheresis, alemtuzumab
Inhibition of complement	IVIG, eculizumab, C1 esterase inhibitors
Inhibition of IL-6	Tocilizumab, clazakizumab

DnDSA: de novo donor-specific antibody; IVIG: intravenous immunoglobulin; ATG: antithymocyte globulin.

## Data Availability

Not applicable.

## Abbreviations

ATG	Antithymocyte globulin
AMR	Antibody-mediated rejection
CAV	Cardiac allograft vasculopathy
DSA	Donor-specific antibody
dd-cfDNA	Donor-derived cell-free DNA
dnDSA	De novo DSA
HLA	Human Leukocyte Antigen
HT	Heart transplant
ISHLT	International Society for Heart and Lung Transplantation
IVIG	Intravenous immunoglobulin
MFI	Mean fluorescence intensity
mTOR	Mammalian/mechanistic target of rapamycin
MICA/B	MHC class I polypeptide-related sequence A/B
SAB	Single-antigen bead
